# Genome-resolved metagenome and metatranscriptome analyses of thermophilic composting reveal key bacterial players and their metabolic interactions

**DOI:** 10.1186/s12864-021-07957-9

**Published:** 2021-09-10

**Authors:** Lucas Palma Perez Braga, Roberta Verciano Pereira, Layla Farage Martins, Livia Maria Silva Moura, Fabio Beltrame Sanchez, José Salvatore Leister Patané, Aline Maria da Silva, João Carlos Setubal

**Affiliations:** 1 Departamento de Bioquímica, Instituto de Química, Universidade de São Paulo, São Paulo, Brazil; 2 Programa de Pós-Graduação Interunidades em Bioinformática, Universidade de São Paulo, São Paulo, Brazil; 3 Laboratório Especial de Ciclo Celular, Instituto Butantan, São Paulo, SP Brazil

**Keywords:** Metagenome-assembled genome, Microbiome, Biomass degradation, *Rhodothermus marinus*, *Thermobispora bispora*

## Abstract

**Background:**

Composting is an important technique for environment-friendly degradation of organic material, and is a microbe-driven process. Previous metagenomic studies of composting have presented a general description of the taxonomic and functional diversity of its microbial populations, but they have lacked more specific information on the key organisms that are active during the process.

**Results:**

Here we present and analyze 60 mostly high-quality metagenome-assembled genomes (MAGs) recovered from time-series samples of two thermophilic composting cells, of which 47 are potentially new bacterial species; 24 of those did not have any hits in two public MAG datasets at the 95% average nucleotide identity level. Analyses of gene content and expressed functions based on metatranscriptome data for one of the cells grouped the MAGs in three clusters along the 99-day composting process. By applying metabolic modeling methods, we were able to predict metabolic dependencies between MAGs. These models indicate the importance of coadjuvant bacteria that do not carry out lignocellulose degradation but may contribute to the management of reactive oxygen species and with enzymes that increase bioenergetic efficiency in composting, such as hydrogenases and N_2_O reductase. Strong metabolic dependencies predicted between MAGs revealed key interactions relying on exchange of H^+^, NH_3_, O_2_ and CO_2_, as well as glucose, glutamate, succinate, fumarate and others, highlighting the importance of functional stratification and syntrophic interactions during biomass conversion. Our model includes 22 out of 49 MAGs recovered from one composting cell data. Based on this model we highlight that *Rhodothermus marinus, Thermobispora bispora* and a novel Gammaproteobacterium are dominant players in chemolithotrophic metabolism and cross-feeding interactions.

**Conclusions:**

The results obtained expand our knowledge of the taxonomic and functional diversity of composting bacteria and provide a model of their dynamic metabolic interactions.

**Supplementary Information:**

The online version contains supplementary material available at 10.1186/s12864-021-07957-9.

## Background

Thermophilic composting is carried out by microbial communities that are able to thrive in this harsh environment [1, 2]. Recent studies have demonstrated that composting microbiomes comprise an enormous diversity of mesophilic and thermophilic microorganisms depending on the method and conditions as well as the stage of the composting process [1–5]. Composting microbes present a remarkable metabolic flexibility and are efficient in breaking down complex organic matter such as lignocellulosic biomass [1, 4, 6].

Lignocellulosic biomass is composed by different biopolymers: cellulose (25–55%), hemicellulose (19–40%), lignin (18–35%), and smaller fractions of pectin and minerals. Therefore, a diverse set of enzymes is required for effective saccharification [7]. Microbial dynamics during lignocellulose breakdown seems to be heavily dependent on syntrophic interactions [8]. The microbial populations need to share the burden of enzymatic production; and sharing metabolites reduces the negative feedback effect of intermediate metabolite accumulation [9]. Syntrophic interactions can involve opportunistic microbes in biomass degrading systems, which are bacteria that do not express or very often lack the required enzymes for biomass degradation, but constitute one important portion of the microbial community, being referred to as ‘sugar cheaters’ [8].

The composting microbiome is considered a valuable microbial resource for biomass degradation, with potential for contributing to a number of biotechnological applications besides its remarkable activities on soil bioremediation and suppressiveness against plant diseases [2, 6, 10, 11]. In spite of this potential, knowledge on how to control and explore those microbes and their functions remains encrypted within their genomes and the multiple combinations of metabolic pathways that they can activate [6, 8, 12]. Research on composting microbes has focused mainly on enriched cultures [8, 13, 14] or taxonomic biodiversity assessments based on 16S rRNA gene amplicon sequencing data [3, 5]. Yet, these methods cannot fully assess microbial functional diversity and metabolic activity.

Shotgun metagenomic sequencing has helped to reveal the diversity of microbial communities in natural habitats [15] and in engineered environments such as composting [1, 16] or sludge digesters [17]. New methods and computational tools now allow the recovery of metagenome-assembled genomes (MAGs) from complex ecosystems [18–23]. The study of MAGs from microorganisms in a given environment can provide detailed taxonomical and functional diversity information, and therefore has the potential to allow a better understanding of their ecological context and metabolic arsenal [22, 24].

Here we present an analysis of MAGs obtained from time-series samples of a thermophilic composting process. These datasets have been analyzed previously, but not from a MAG perspective [1]⁠. Our aim was to obtain a detailed view of the microbial populations active in a composting process and to determine their metabolic interactions, thus advancing on our previous work [1]. We used the collection of genomes recovered to build a framework for the temporal dynamics of microbial molecular processes during composting. Using this framework as a reference, we built genome-scale metabolic models for predicting syntrophic interactions and the more frequently exchanged compounds.

## Results

### MAGs recovered from thermophilic composting

We recovered a total of 11 and 49 MAGs (Metagenome-assembled genomes), respectively, from metagenomes of ZC3 and ZC4 composting samples (Table 1). All these 60 MAGs (Supplementary Table 1) meet the medium-quality requirement (≥ 50% completeness and ≤ 10% contamination) of the MIMAG standard [25], with the exception of ZC3RG09, which had 10.87% contamination. Thirty-four MAGs meet the high-quality requirement (≥ 90% completeness and ≤ 5% contamination). The average number of contigs in these MAGs is 363.75, with a minimum of 15 (ZC4RG10) and a maximum of 1655 (ZC4RG48) (Supplementary Table 1). The genome size of the 60 MAGs varies from 5.7 Mbp (ZC4RG46) to 1.5 Mbp (ZC4RG49) and their average %GC is 64.21 ± 8.78 (Supplementary Table 1). Pairwise comparisons (Supplementary Table 2) showed that six MAGs recovered from ZC3 metagenomes (ZC3RG05, ZC3RG06, ZC3RG07, ZC3RG08, ZC3RG09 and ZC3RG11) are highly similar (two-way ANI measure ≥99%; DDH estimate ≥95%) to six MAGs recovered from ZC4 metagenomes (ZC4RG21, ZC4RG10, ZC4RG11, ZC4RG09, ZC4RG06 and ZC4RG18, respectively); this level of similarity is what we call ‘MAG redundancy’ further down. These MAGs may represent genomes from different strains from the same bacterial species.

**Table 1:**
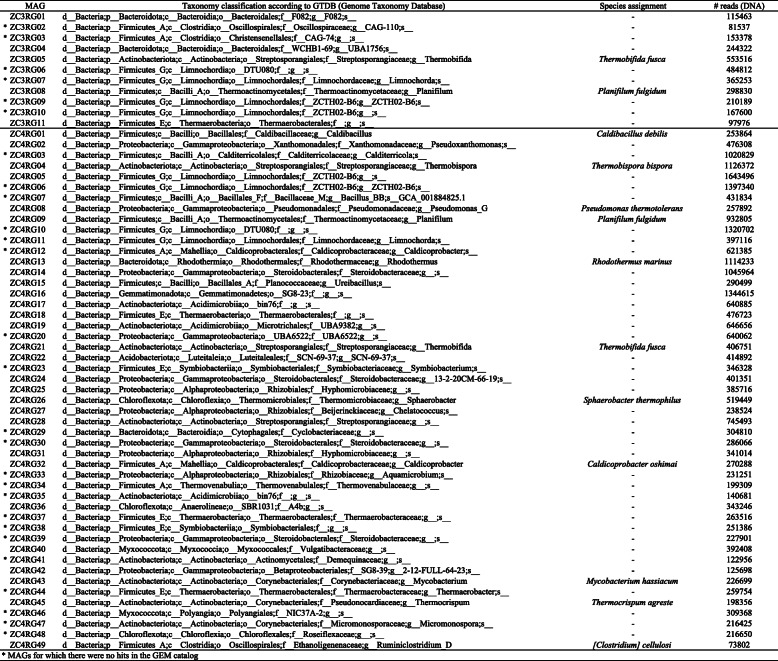
MAGs recovered from ZC3 and ZC4 thermophilic composting

* MAGs for which there were no hits in the GEM catalog

Mapping of ZC4 metagenome reads to ZC4 MAGs shows that the 49 ZC4 MAGs account for 21.8% of all ZC4 reads (the number of ZC4 metagenome reads per MAG is shown in the last column of Table 1; adding that column results in 24,539,668 reads. The total number of ZC4 reads is 112,736,134 [1]). A similar calculation for ZC3 shows that the 11 ZC3 MAGs account for 29.6% of all reads in those samples.

### Taxonomic assignments

The 60 recovered MAGs were assigned to six different phyla: Acidobacteriota, Actinobacteriota, Bacteroidota, Chloroflexota, Firmicutes and Proteobacteria (Table 1) according to GTDB [26]. At the order level there is remarkable diversity, with 32 different orders represented by these MAGs. The most frequent order was Limnochordales (6 MAGs). Most of the ZC3 and ZC4 MAGs seem to be novel: there are eight MAGs for which no family could be assigned, 18 MAGs for which no genus could be assigned, and 17 MAGs for which no species could be assigned; this takes into consideration the “redundancy” in MAGs between ZC3 and ZC4.

Thirteen MAGs could be assigned to 11 species (Table 1) for which there is at least one isolate genome publicly available in the NCBI RefSeq repository (Supplementary Table 3). Pairwise genome comparisons showed that in all these cases the two-way ANI measure was at least 98% and the DDH estimate was at least 87% (Supplementary Table 3), strongly suggesting that the assignments are correct and confirming that the recovered MAGs are of high quality. Several of the 11 species to which these 13 MAGs were assigned are known as thermophilic bacteria: *Thermobifida fusca*, *Thermobispora bispora*, *Pseudomonas thermotolerans*, *Rhodothermus marinus*, and *Planifilum fulgidum*. Except for *Mycobacterium hassiacum,* which has been isolated from human samples, the other 10 species have been found in environments related to biomass degradation, such as compost, decaying wood, and animal feces (Supplementary Table 4).

### ZC3 and ZC4 MAGs in other environments

**Fig. 1:**
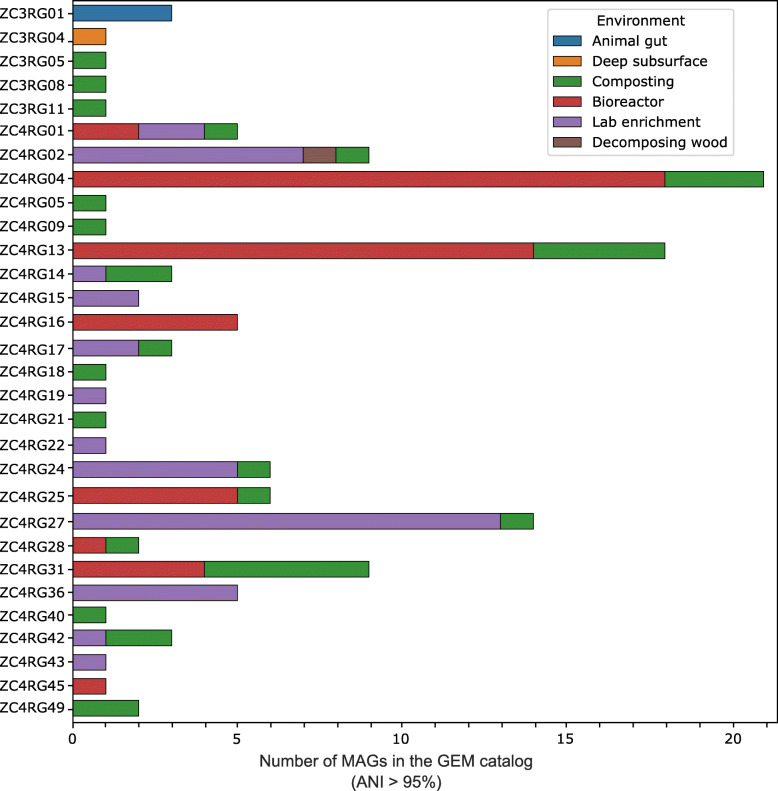
Hits of the 60 composting MAGs in the Genomic catalog of Earth’s Microbiomes (GEM), using as threshold average nucleotide identity equal to or greater than 95%; details in Supplementary Table 5

We checked for the presence of our 60 MAGs in two publicly available MAG datasets [22, 23]. The recovery of the “same” genome from different environments lends additional confidence to our MAG recovery process. Among the 910 MAGs from the Asian soil, plant-based compost, and leafy greens phytobiomes [23] we found no relevant hits (i.e., ANI was less than 85%). We did find hits (ANI ≥ 95%) for 30 of our ZC3/ZC4 60 MAGs in the Genomes from the Earth’s Microbiomes (GEM) catalog [22] (Fig. 1, Supplementary Table 5). Genomes similar to these 30 MAGs were mostly recovered from environments associated with biomass-degradation, including cellulose-adapted laboratory enrichments and composting environments, as well as animal (capybara and moose guts), deep subsurface shale carbon reservoir, and bioreactor metagenomes (Fig. 1), suggesting that these bacterial populations are highly specific to biomass-degrading environments (Fig. 1, Supplementary Table 5). ZC4RG04 (*Thermobispora bispora*) and ZC4RG13 (*Rhodothermus marinus*) were the MAGs with most hits in the GEM catalog (21 and 18 hits, respectively). With this analysis we observed that some MAGs co-occurring in the Zoo composting were also co-occurring in other environments. For instance, GEM MAGs corresponding to ZC4RG05 (Limnochordales), ZC3RG11/ZC4RG18 (Thermaerobacterales), and ZC3RG08/ZC4RG09 (*Planifilum fulgidum*), were also recovered from a steer manure compost metagenome dataset (Supplementary Table 5).

Additional comparisons (not shown) revealed that ZC4RG01 (*Caldibacillus debilis*) and ZC3RG09/ZC4RG06 are similar (ANI ≥ 99%), respectively, to genomes BZ5 and BZ6, which are two MAGs recovered from a thermophilic compost-derived consortium named ZCTH02 [13]. The composting facility from which this consortium was obtained is the same that provided the 60 MAGs here studied. ZC3RG09/ZC4RG06, ZC3RG10, and ZC4RG05 have been assigned by the GTDB group to a provisionally named family called ZCTH02-B6, in the order Limnochordales (Table 1).

Based on the results above, in our MAG dataset there are 24 nonredundant MAGs that have no species assigned and have no hits in the two MAG catalogs against which we searched, and therefore represent truly novel contributions to known MAG diversity.

### Functional analysis of composting MAGs

For functional analysis, we have focused on ZC4 MAGs. Our rationale was that only for ZC4 samples do we have metatranscriptome data. These RNA-seq datasets were obtained for eight time-series samples (days 1, 3, 7, 15, 30, 64, 78 and 99 of composting) as previously reported [1]. The temperature of ZC4 composting cell at the day of sample collection varied from 66.2 °C (day 1) to 47.8 °C (day 99), being around 65–70 °C most of the time, as detailed in Table 1 of Antunes et al. [1]. The relative abundance in transcripts per kilobase million reads (TPM) for each one of the 49 MAGs over time showed that all of them were transcriptionally active (Supplementary Table 6).

### Lignocellulose degradation

**Fig. 2:**
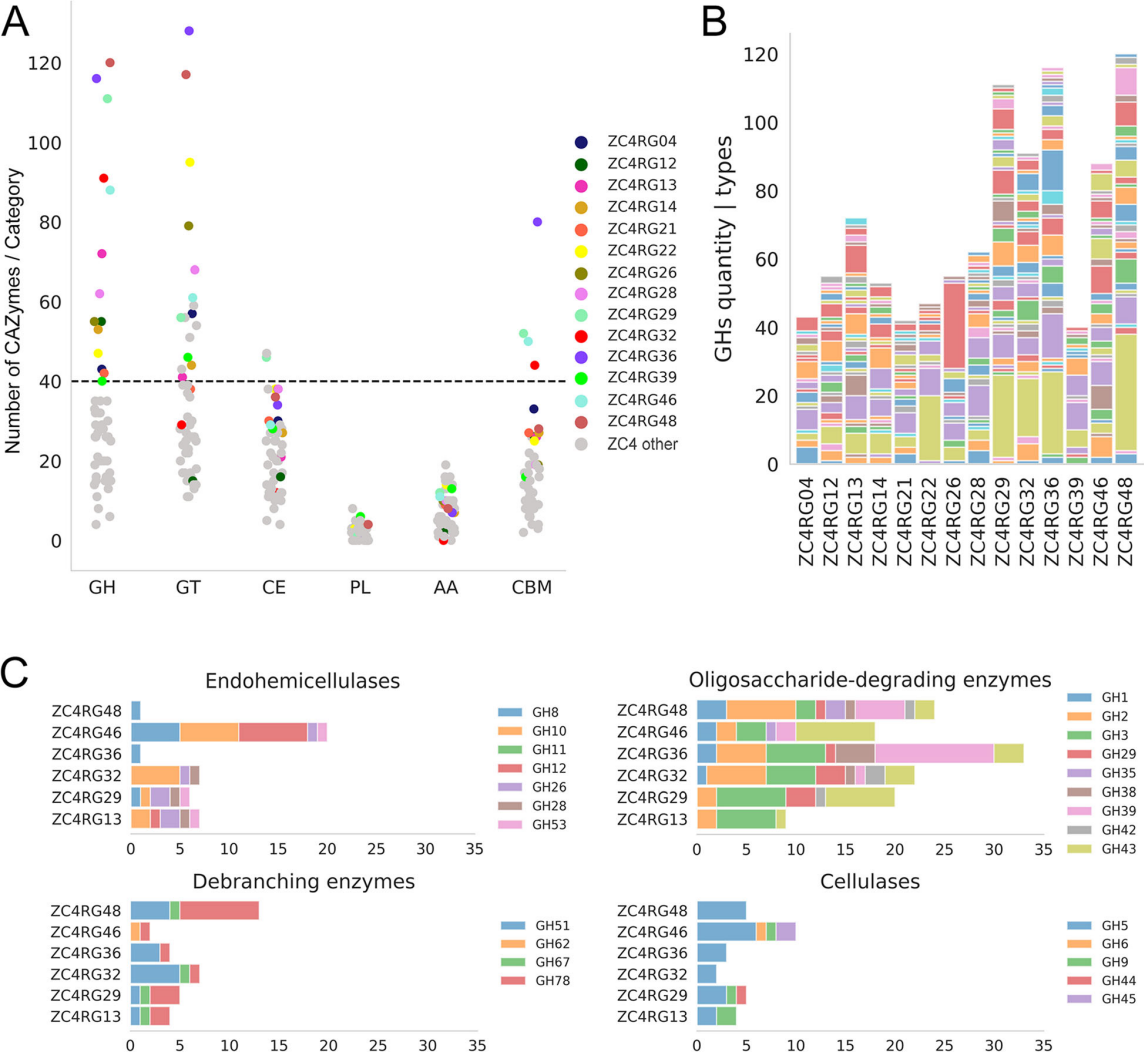
Metabolic potential of MAGs from the ZC4 composting cell based on CAZymes. **a** CAZyme Genes in 14 MAGs with at least 40 Genes annotated as GHs (dashed line). **b** Breakdown of GH families for the same 14 MAGs as in (**a**). **c** Comparison of numbers of Genes annotated as GHs related to lignocellulose degradation in the top six degraders (ZC4RG13, ZC4RG29, ZC4RG32, ZC4RG36, ZC4RG46, and ZC4RG48)

Biomass degrading capabilities in MAGs were analyzed based on COG (Clusters of Orthologous Groups) assignments (Supplementary Fig. 1) and CAZy annotations (Supplementary Tables 7 and 8; Fig. 2) of their respective genes. Among the 49 ZC4 MAGs, 26 encode more than 100 genes classified as CAZymes (Supplementary Table 7). Out of these, 14 MAGs encode at least 40 genes classified as GHs (Glycoside Hydrolases) (Fig. 2a; Supplementary Table 7). Several cellulases (GH5, GH6, GH9 and GH45), endohemicellulases (GH8, GH10, GH11, GH12, GH26, GH28 and GH53), debranching (GH51, GH62, GH67 and GH78) and oligosaccharide-degrading enzymes (GH1, GH2, GH3, GH29, GH35, GH38, GH39, GH42 and GH43) were annotated in these MAGs (Fig. 2b and c). The categorization of GHs just presented follows the categorization of the CAZyme database [27].

Regarding Auxiliary Activities (AA), ZC4RG20 (Gammaproteobacteria), ZC4RG33 (*Aquamicrobium*), ZC4RG43 (*Mycobacterium hassiacum*), and ZC4RG45 (*Thermocrispum agreste*) present at least 15 genes classified as AA (Supplementary Table 7). ZC4RG45 contains the highest diversity of AA genes (Supplementary Table 8). Members of the AA1 family, which perform lignin degradation efficiently, were only found in ZC4RG08 (*Pseudomonas thermotolerans*) (Supplementary Table 8). ZC4RG21 (*Thermobifida fusca*), ZC4RG04 (*Thermobispora bispora*), ZC4RG28 (Streptosporangiaceae), ZC4RG45, and ZC4RG47 (*Micromonospora*) were the only ones containing genes classified in the AA10 family (lytic polysaccharide monooxygenases) (Supplementary Table 8), members of which are capable of directly targeting cellulose for oxidative cleavage of the glucose chains.

**Fig. 3:**
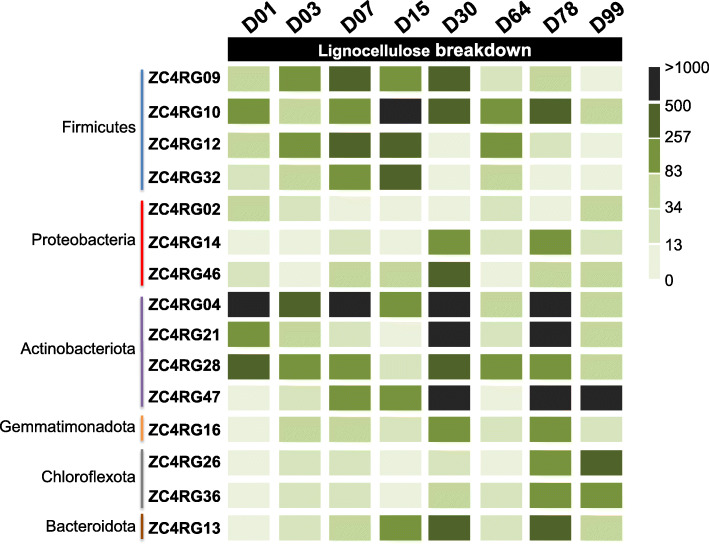
Heatmap representing the abundance of transcripts associated with genes annotated with functions related to lignocellulose degradation in the indicated MAGs. The scale in shades of green is based on relative abundance (Transcripts per kilobase Million Reads) obtained from data shown in Supplementary Table 9

As stated above, we have strong evidence that each ZC4 MAG here analyzed was transcriptionally active during composting. We checked the expression of genes related to lignocellulose degradation and determined that all CAZymes mentioned had corresponding transcripts in the ZC4 metatranscriptome dataset, and their abundance varies over time (Fig. 3 and Supplementary Table 9).

### Secondary metabolites

**Fig. 4:**
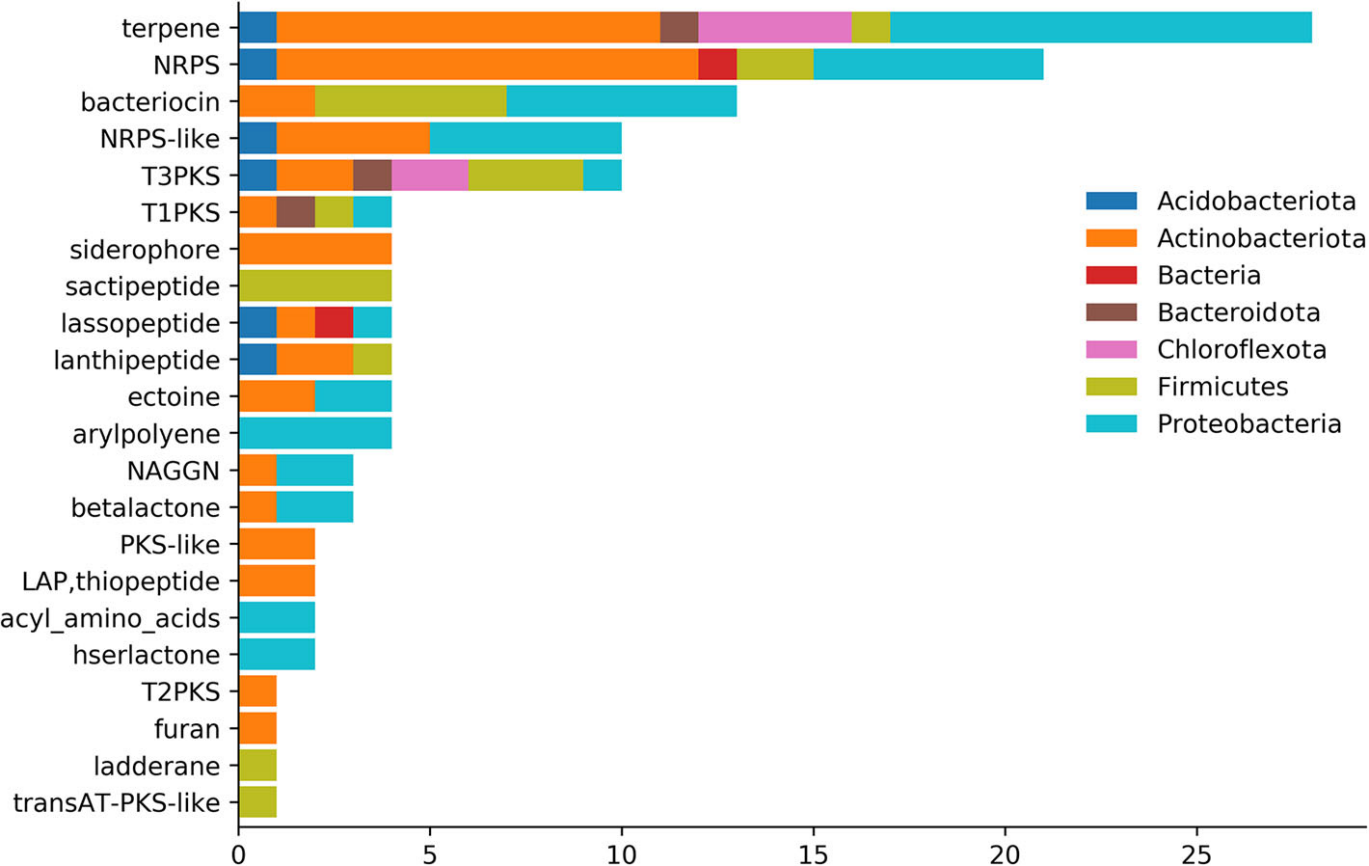
Secondary metabolite cluster types detected among the ZC4 MAGs. The X-axis represents the number of clusters detected. The colors in the bars correspond to phylum assignment of MAGs

Several coding sequences classified as secondary metabolite genes (such as siderophores, bacteriocins, sacpeptides, betalactones, lassopeptides, and type I, II and III polyketides) were found in MAGs (Fig. 4 and Supplementary Table 10). MAGs with at least 100 genes annotated as secondary metabolites were: ZC4RG04 (*Thermobispora bispora*), 121 genes; ZC4RG21 (*Thermobifida fusca*), 105 genes; ZC4RG22 (Luteitaleales), 115 genes; and ZC4RG39 (Steroidobacteraceae), 100 genes. (The MAG with the fifth largest repertoire of secondary metabolite genes had 58.) Transcripts for secondary metabolite genes were generally more abundant at the beginning of the composting process (days 1, 3, and 7) than in the later stages (days 78 and 99) (Supplementary Table 10). The range in TPM values greater than zero per gene per sample day was quite large: from near zero to 2162, with the vast majority (95.46%) being less than 50. We can therefore define a highly expressed (HE) gene as secondary metabolism gene with at least 50 TPM in a sample day. Using this definition, the following MAGs stand out: ZC4RG04 (*T. bispora*) had 34 HE genes on days 1 and 3, the most of any MAG, being the activity level on other days much smaller; ZC4RG03 (*Calditerricola*) and ZC4RG07 (*Bacillus*) had the most HE genes in days 1, 3, 7, 15, and 64: 47 for ZC4RG03 and 32 for ZC4RG07. The peak day, however, was different: for ZC4RG07 it was on day 3 (13 HE genes), and for ZC4RG03 it was on day 15 (14 HE genes). For ZC4RG03 moreover, expression of any secondary metabolite gene was essentially zero on days 30, 78, and 99. ZC4RG21 (*T. fusca*) was by far the MAG with most HE genes on day 30 (15 HE genes). The gene with the maximum TPM value (2162.5) belongs to ZC4RG03 (*Calditerricola*) and was annotated as a plantaricin C family lantibiotic (locus tag C0P64_01260). It is interesting to note that the two adjacent and upstream genes were also annotated as secondary metabolite genes (lantipeptide synthetase LanM and bacitracin ABC transporter ATP-binding protein), and they were also HE.

### Antibiotic resistance genes

Antibiotic resistance genes (ARGs) were observed mainly in MAGs from Actinobacteriota and Proteobacteria phyla (Supplementary Table 11). ZC4RG08 (*Pseudomonas thermotolerans*) has the largest number of expressed ARGs, with 12 genes encoding multidrug efflux pumps (MuxABC-OpmB, MexAB-OprM, MexEF-OprN, MexWV, and MexJK). The MAG that is ranked second in terms of number of expressed ARGs is ZC4RG43 (*Mycobacterium hassiacum*), with seven genes; all other MAGs express five or fewer ARGs (Supplementary Table 11). Among frequently detected ARGs, two genes may be remarked, because each of them is expressed by seven MAGs (out of 15): *rpoB2*, which encodes a rifampicin-refractory beta-subunit of RNA polymerase [28], and *mtrA*, which encodes the MtrCDE multidrug efflux pump transcriptional activator [29] (Supplementary Table 11). Overall, transcripts of ARGs were more abundant in days 1, 3, 30 and 78 of the composting process (Supplementary Table 11).

### Aerobic and anaerobic respiration strategies

The analysis of oxygen metabolism indicates that 45 out 49 bacteria from which MAGs were obtained are aerobes (Table 2). All the oxidase genes annotated in the ZC4 MAGs are predicted to be active and their transcript abundance variation over time shows a slight decrease after day 7, with an increase following day 64 (immediately after the turning procedure of the composting cell) (Supplementary Fig. 2). Evidence of oxidases and aerobic metabolism was not detected in MAGs ZC4RG12 (*Caldicoprobacter*), ZC4RG32 (*C. oshimai*), ZC4RG34 (Thermovenabulaceae), and ZC4RG49 (*[Clostridium] cellulosi*), thereby indicating a metabolism strictly anaerobic. These four MAGs have been classified within the phylum Firmicutes and had an activity profile (based on the global abundance variation of their transcripts over time) with a peak in the early stages of composting (day 3 to 15) (Supplementary Table 6).

**Table 2:**
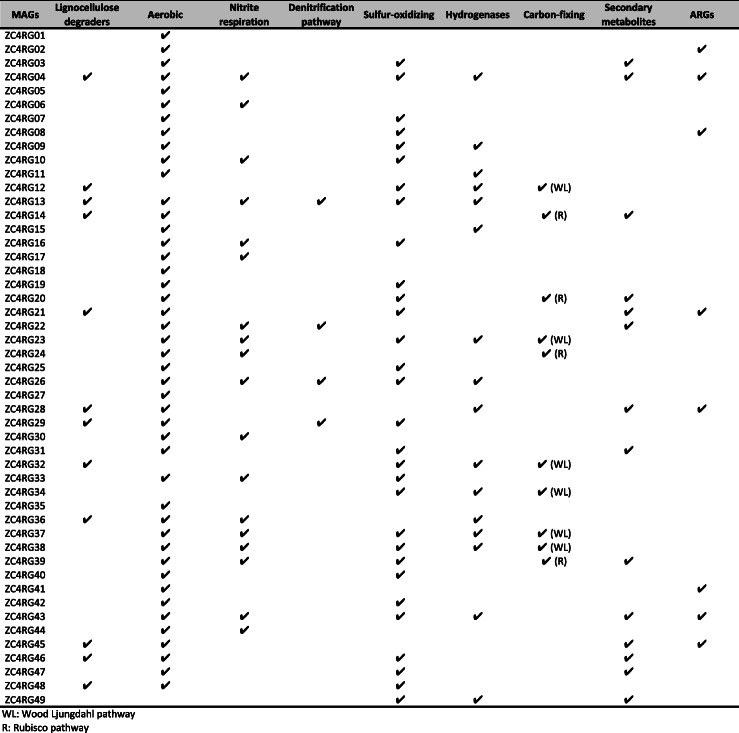
Metabolic profile of ZC4 MAGs. ARGs: Antibiotic Resistance Genes

*WL* Wood Ljungdahl pathway, *R* Rubisco pathway

Evidence for dissimilatory sulfite reductase function (*dsr*AB and *aps*A) was not detected in the genomes, suggesting that the respiratory sulfate reduction was not the main strategy for anaerobic respiration employed by the MAGs here studied.

Active denitrification genes were detected in several MAGs (Table 2). Eighteen of them presented the nitrite respiration gene *nir*K. The variant *nir*S was not detected in the MAGs. ZC4RG13 (*Rhodothermus marinus*), ZC4RG22 (Luteitaleales), ZC4RG26 (*Sphaerobacter thermophilus*), and ZC4RG29 (Cyclobacteriaceae) encode the complete denitrification pathway (i.e., nitrous-oxide reductase pathway, *nos*ZD). These MAGs also have genes that code for *nir*B instead of *nir*K for nitrite reductase (Table 2). Nitrous-oxide reductase genes (*nos*ZD) were also active during the composting process. The variation in abundance of transcripts of these denitrification genes increased over time, with a peak in day 7 (Supplementary Fig. 2).

### Chemolithotrophic metabolism

We found evidence for chemolithotrophic metabolism based on MAG genes related to the oxidation of inorganic sulfur compounds (Table 2). Nearly all MAGs have genes annotated with products from the sulfur oxidation pathway via sulfur dioxygenase. Some of the MAGs have genes annotated with other functions associated with the oxidation of sulfur compounds. ZC4RG20 (Gammaproteobacteria), for instance, represents a bacterial population that showed transcripts associated with sulfur dioxygenase, sulfide oxidation (sqr), and thiosulfate oxidation (*sox*C), including transcripts associated with carbon fixation via rubisco activation, supporting a chemolithoautotrophic growth. The Sox system, which is able to oxidize sulfite and sulfone group in thiosulfate, was found in MAGs ZC4RG25 (Hyphomicrobiaceae), ZC4RG31 (Hyphomicrobiaceae), ZC4RG33 (*Aquamicrobium*), and ZC4RG42 (Betaproteobacteria), although *sox*C was apparently lacking in all of them. Sulfide oxidation (sulfide- quinone reductase, sqr) to thiosulfate was detected also in ZC4RG25. These observations highlight that members of the bacterial populations in the composting microbiome were capable of harvesting energy by oxidizing inorganic sulfur compounds. Coding sequences annotated as nitrification genes (*amo* and *hao*) were not detected in the ZC4 MAGs, indicating that this trait was of minor or no relevance for the composting microbiome.

Several hydrogenases were found to be present and expressed (Table 2, Supplementary Fig. 2). We were able to identify two types of hydrogenases. MAGs ZC4RG04, 09, 13, 15, 26, 28, 36, 37, 38, 43, and 49, belonging to diverse phyla (Table 1), have genes annotated as [NiFe] hydrogenases. MAGs ZC4RG11, 12, 23, 32, 34, 38, and 49, from the phylum Firmicutes (Table 1), have genes annotated as prototypical hydrogen-evolving [FeFe] hydrogenases (group A1).

### Correlation of MAGs based on their activity profiles

**Fig. 5:**
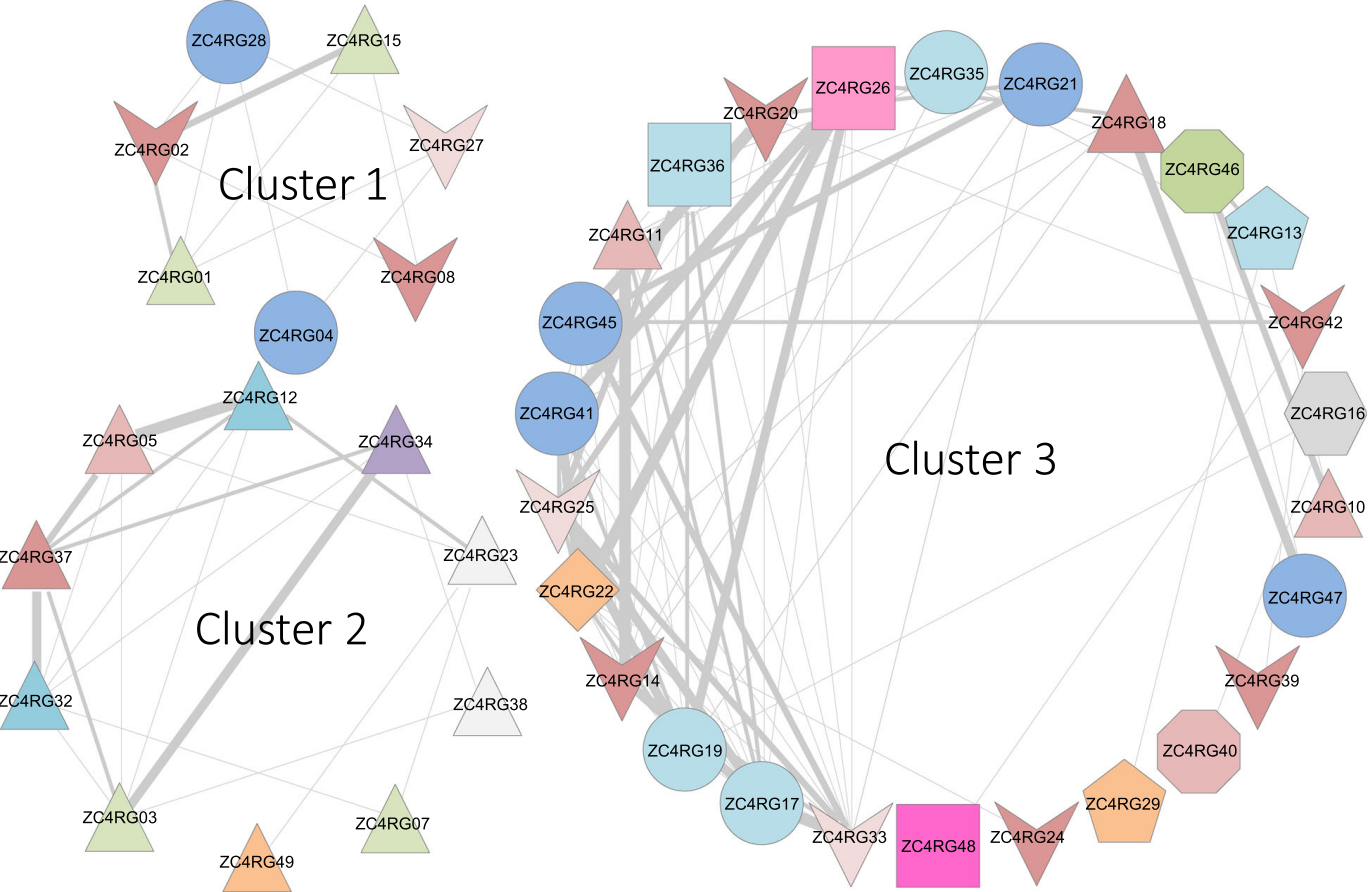
MAGs co-occurrence based on their relative abundance in metatranscriptomes of the ZC4 composting cell. Nodes represent MAGs and edges represent Spearman correlations (r^2^ ≥ 0.8), in the following five intervals: ≥ 0.97; (0.97..0.95]; (0.95..0.92]; (0.92..0.90], and less than 0.90. (For actual pairwise values, see Supplementary Table 12.) Different shapes indicate different phyla: Acidobacteriaota (diamond), Actinobacteriota (circle), Bacteroidota (pentagon), Chloroflexota (square), Firmicutes (triangle), Gemmatimonadota (hexagon), Myxococcota (octagon), and Proteobacteria (inverted triangle). Colors indicate different classes within a phylum (see Table 1)

Using the 49 ZC4 MAGs, we computed correlation patterns using their variation in activity during the composting process inferred from ZC4 metatranscriptomic data. The correlation patterns derived from the activity profile resulted in a graph composed by 43 nodes and 76 interactions, and three clusters (Fig. 5). In what follows we describe the main features of each activity cluster, highlighting days when a relatively high number of transcripts associated with specific MAGs in each cluster was observed (Supplementary Table 6).

#### Cluster 1

Seven MAGs form this cluster. Transcripts from members of this cluster are more abundant on initial days of composting (days 1 and 3) with a slight later increase on day 64 (immediately after turning), followed by another increase on day 99. Cluster members ZC4RG02 (*Pseudoxanthomonas*), ZC4RG04 (*Thermobispora bispora*), and ZC4RG28 (Streptosporangiaceae) presented the highest number of transcripts in the initial days.

#### Cluster 2

This cluster is composed of 10 MAGs, all Firmicutes and mostly abundant and active between days 3 and 15, followed by a peak on day 64. Many transcripts from ZC4RG12 (*Caldicoprobacter*) and ZC4RG32 (*C. oshimai*) related to lignocellulose degradation were identified, especially on days 3, 7, and 15.

#### Cluster 3

This 26-MAG cluster is taxonomically diverse (it contains members of Acidobacteriota, Actinobacteriota, Bacteroidota, Chloroflexota, and Proteobacteria phyla). The following cluster members are notable for expressing genes related to lignocellulose breakdown: ZC4RG13 (*Rhodothermus marinus*), ZC4RG14 (Steroidobacteraceae), ZC4RG21 (*Thermobifida fusca*), ​​ZC4RG26 (*Sphaerobacter thermophilus*), ZC4RG29 (Cyclobacteriaceae), ZC4RG36 (Anaerolinea), ZC4RG46 (Polyangiales), ZC4RG47 (*Micromonospora*), and ZC4RG48 (Roseiflexaceae); this activity is especially intense on days 30, 78, and 99. ZC4RG20 (Gammaproteobacteria) and ZC4RG45 (*Thermocrispum agreste*) express several genes associated with lignin degradation (i.e., annotated with CAZy AA families), especially on day 99.

### Metabolic dependencies based on genome-scale models

**Fig. 6:**
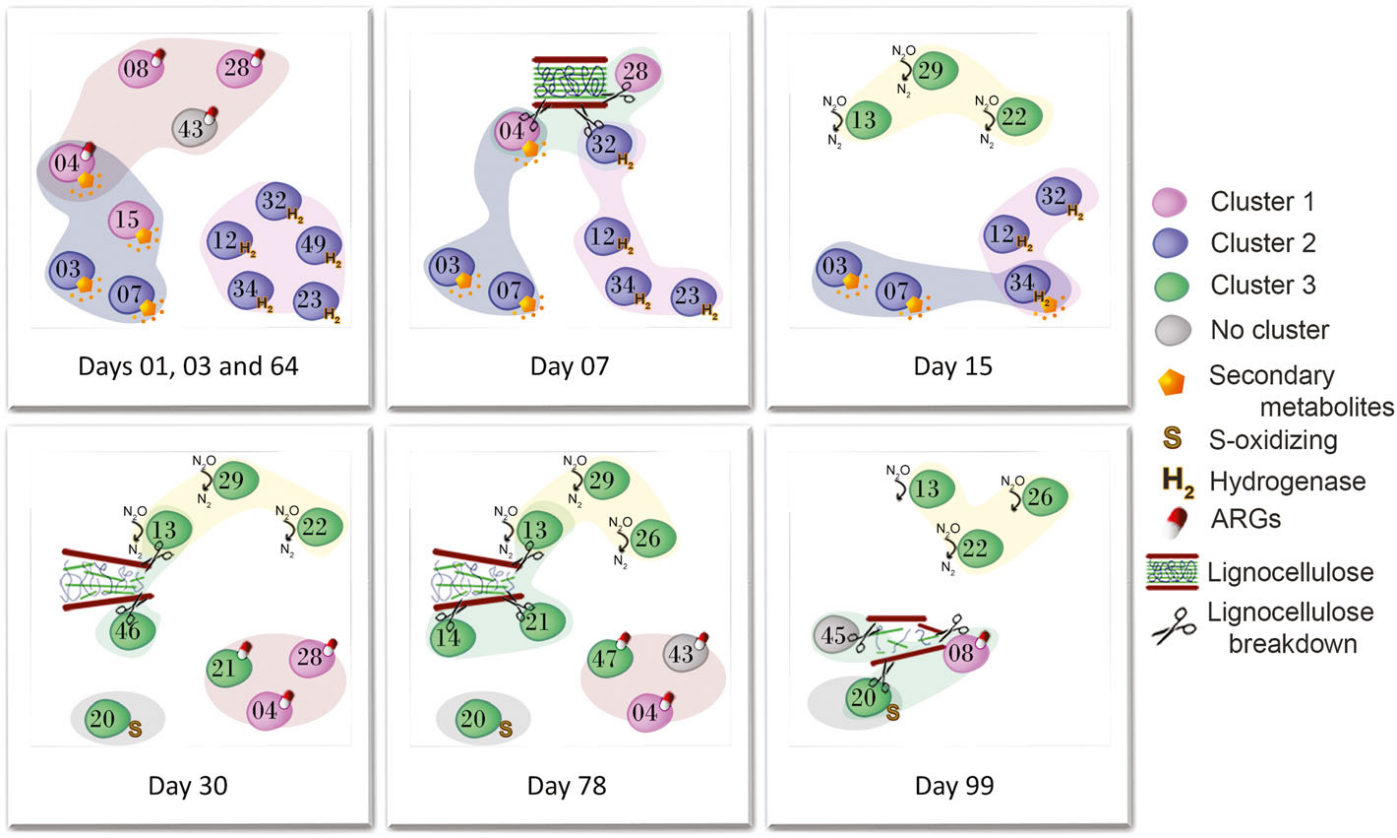
Schematic representation of keystone microbial players according to their importance in the different stages of composting. MAGs are represented as roughly circular numbered shapes, and their colors reflect the cluster they were assigned to (Fig. 5 and Supplementary Table 4). Lignocellulose breakdown and relevant active functions are represented across the stages by the various symbols, and the irregular background shapes connect MAGs that express the same functions. The turning procedure was performed on day 63, therefore day 64 is considered a recapitulation of the start of composting (days 1 and 3), based on the patterns of microbial functions and activity that we observed in the present study and in our previous work [1]⁠

Based on the results obtained with the correlation analysis using the transcriptional activity profile of MAGs (Fig. 5, Supplementary Table 6, Supplementary Table 12) and the activity of relevant genes (Table 2, Supplementary Tables 9, 10, 11, Fig. 3, and Supplementary Fig. 2), we identified MAGs according to their importance in the different stages of composting and the main functions associated with them (Fig. 6). We then used this framework to assess the metabolic dependencies between these MAGs based on genome-scale models. The results revealed strong dependencies (i.e. the maximum dependency score = 1) between some of the MAGs (Supplementary Table 13). According to the models obtained, the most frequent compounds involved in the interactions between MAGs are H^+^, NH_3_, O_2_, CO_2_, as well as glutamate, fumarate, succinate, glucose, and hypoxanthine (Supplementary Table 13). The top three MAGs with highest number of interactions for metabolic exchange were ZC4RG13 (*Rhodothermus marinus*), ZC4RG04 (*Thermobispora bispora*) and ZC4RG20 (Gammaproteobacteria). ZC4RG13 interactions were mostly as a compound donor, while ZC4RG04 and ZC4RG20 interactions were mostly as compound receivers (Supplementary Table 14A).

To test if the predicted metabolic interactions are likely to be specific of the bacterial strains that were able to thrive in the composting microbiome, we reran the metabolic interaction analysis replacing the MAGs assigned to known species by the respective reference genomes from GenBank according to the taxonomic assignment (Table 1). We found that ZC4RG04, ZC4RG13 and ZC4RG20 were no longer classified among the top three genomes with highest number of interactions in the model (Supplementary Table 14B).

## Discussion

Sixty MAGs were recovered, of which 47 are potentially from new bacterial species and 24 represent novel genomes. Thirty-three of our MAGs had good matches to GEM MAGs (30) or to isolates (3) from environments in geographical areas distinct from where we got our samples. This fact shows that these MAGs (and by extrapolation, all MAGs in our dataset) are not artifacts, and are good approximations (as all MAGs are) to the genomes of the bacterial species living in these environments. It also suggests that similar environments, even if geographically very distant, have a tendency to select the same bacterial species. There may be some species that are specific to the environment we studied, but this will need to be rechecked in the future, as more MAGs from different parts of the world become available.

We focused our functional analysis on the 49 MAGs that were retrieved from the ZC4 composting cell. One may ask to what extent these 49 MAGs are representative of the composting process. We can answer this question by referring to our previous results, based on the same baseline datasets, and that took into account all shotgun sequencing reads [1]. At the phylum level, that work stated that Firmicutes, Proteobacteria, Bacteroidota and Actinobacteriota accounted for at least 85% of all classified reads in all samples. A breakdown of the 49 ZC4 MAGs in terms of phylum shows that those four phyla account for 86% of these 49 MAGs, therefore in close agreement with the previous result. At deeper taxonomic levels, the comparison becomes more difficult, primarily because read-based classification is still unreliable [30, 31]. On the other hand, the 49 ZC4 MAGs account for 21.8% of all metagenome reads. Based on these results, we believe that the MAG-based functional results we present are representative of key microbial processes taking place in this thermophilic composting, while certainly very far from exhausting the tremendous functional diversity of this environment.

Among the recovered genomes, ZC4RG01 (*Caldibacillus debilis*), ZC4RG04 (*Thermobispora bispora*), ZC4RG13 (*Rhodothermus marinus*), ZC4RG21 (*Thermobifida fusca*), ZC4RG26 (*Sphaerobacter thermophilus*), ZC4RG32 (*C. oshimai*), and ZC4RG49 ([*Clostridium*] *cellulosi*) have been classified as species previously reported as being capable of cellulose degradation [13, 32–37]⁠. Two Chloroflexota MAGs, ZC4RG36 (Anaerolineae) and ZC4RG48 (Roseiflexaceae), are additional lignocellulose degraders that we have found, having many genes classified as CAZymes (326 and 313, respectively), exceeding the number of CAZymes in the much better-known lignocellulose degraders *Thermobispora bispora* [38] and *Thermobifida fusca* [35], corresponding to ZC4RG04 and ZC4RG21, with 174 and 150 CAZymes, respectively. Chloroflexota bacteria have been reported in biomass-degrading environments using cultivation-independent methods, in some cases associated with the maturing phase of the composting process [39].

We focused our functional analysis on the following processes: lignocellulose degradation, denitrification, sulfur metabolism, hydrogen metabolism, oxygen metabolism, and secondary metabolite and antibiotics production. The need to focus on lignocellulose degradation is obvious given the nature of the samples. For the other processes, our justification is as follows. Denitrification: it is a facultative respiratory biochemistry pathway mediated by denitrifying microbes, which convert nitrate to nitrogen gas or nitrous oxide under strict anaerobic conditions, and constitutes one of the main branches of nitrogen cycle during composting [40]. Sulfur metabolism and hydrogen metabolism: during the thermogenic phase, degradation and mineralization of complex organic matter also take place by autotrophic sulfur oxidizers, which oxidize (and, thus, detoxify) the hydrogen sulfide generated by the mineralization of organic sulfur compounds, whereas hydrogen-oxidizing bacteria use the molecular hydrogen produced by fermentative reactions [41]. Oxygen metabolism: organic matter is composted both aerobically and anaerobically, but aerobic composting is the most efficient form of decomposition and produces finished compost in the shortest time. During the initial stages of composting, the oxidation of organic material by microbial populations, which increases temperature, is at its most intense, but this form of degradation goes on essentially all the time. Finally, secondary metabolite production and antibiotic resistance genes were also analyzed, given their crucial role in microbial interactions, which to a large extent drive the whole process.

We carried out a correlation analysis of MAGs based on their activity profiles during the process. Based on all these results, we propose here a framework for the temporal dynamics of microbial processes in the composting system we have studied (Fig. 6).

MAGs from clusters 1 and 2 (Fig. 5) are the main constituents of the composting stage characterized by days 1, 3 (composting start), and 64 (recapitulation of composting start after the turning procedure) (Fig. 6). These MAGs represent bacterial populations expressing ARG genes and genes with functions related to secondary metabolite production. These activities could be explained by intense competition between microorganisms. Indeed, these stages have high microbial diversity [1]. According to this interpretation, secondary metabolite production and ARG expression would be the consequence of the arms-shields race hypothesized to take place in the composting microbial community [42, 43]⁠. Among the MAGs in these clusters, ZC4RG03 (*Calditerricola*) and ZC4RG07 (*Bacillus*), both from cluster 2, stand out in terms of expression of secondary metabolite genes between day 1 and day 15, and on day 64 (Supplementary Table 10). At a more detailed level, ZC4RG03 contains three adjacent highly expressed genes that are orthologous to the first three genes of the Plantaricin C operon in *Lactobacillus plantarum* [44]. Plantaricin C has been shown to inhibit Gram-positive bacteria [45]. Based on this analysis, we hypothesize that ZC4RG03 and ZC4RG07 represent important players in the composting process, by producing relatively high amounts of compounds with selective antimicrobial activity against pathogenic and opportunistic competitor bacteria. At the same time, they also have the genes required to consume easily degradable compounds, which we assume are particularly abundant during the initial composting stages (or right after the turning procedure). A considerable portion of the composting substrate material that was sampled is made of animal feces, and niche protection through antagonistic competition has been observed in gut microbiota [46, 47].

According to our framework (Fig. 6), MAGs from cluster 2 are primarily active between days 1 and 15 (and right after turning) (Supplementary Table 6). All of them are Firmicutes encoding hydrogenases. H_2_-oxidizing bacteria are a group of facultative autotrophs that can use the molecular hydrogen produced during fermentative conversion of organic compounds as an electron donor during transitions from aerobic to anaerobic decomposition, which possibly justifies the relevance of MAGs with hydrogenase activity at this stage. Hydrogenases participate in the mechanism that allows bacteria to store metabolic energy as an electrochemical potential across the membrane via the proton-motive force. H_2_ metabolism can be coupled with CO_2_ as electron acceptor, which allows autotrophic growth via the acetyl coenzyme A (i.e., the Wood pathway) [48], supporting the metabolisms of diverse prokaryotes, including methanogens, aerobic carboxidotrophs, acetogens, sulfate-reducers, and hydrogenogenic bacteria. MAGs ZC4RG12, 32, 34 are in this last group of bacteria as, besides the hydrogenases, they possess the carbon monoxide dehydrogenase (*codh* gene). Methanogens and sulfate reducers were not detected among the recovered MAGs. Thus, although predominantly aerobic, our results suggest that, at this stage of the composting process (between days 1 and 15), microaerophilic habitats favor anaerobic fermentation.

The presence of obligately and facultatively sulfur- and hydrogen-oxidizing bacteria was already reported in hot composts, suggesting that they may play a part in mineralization, and particularly in inorganic sulfur compound oxidation during the thermogenic phase (> 60°) of the composting process [41]. Also, in the present work, sulfur oxidation capability was detected in ZC4RG25, ZC4RG31, ZC4RG33 and ZC4RG42. Together, these observations suggest that degradation and mineralization of complex organic matter takes place.

The late stages (represented by days 30, 78 and 99) are mainly dominated by MAGs from cluster 3 that perform lignocellulose degradation, denitrification, and sulfur oxidation. In these stages there is a decrease in the overall phylogenetic microbial diversity [1]⁠. Nevertheless, cluster 3 contains the largest and most diverse group of MAGs. We hypothesize that in these stages most nutrients derive from recalcitrant material (e.g., lignin). ZC4RG20 (Gammaproteobacteria) is a cluster 3 MAG with a large repertoire of enzymes classified as Auxiliary Activities, when compared to other MAGs. One MAG that does not belong to cluster 3 but nevertheless seems to be particularly active around day 99 is ZC4RG08 (*Pseudomonas thermotolerans)* (cluster 1). It is the only one to have genes annotated as belonging to the AA1 family. AA1 enzymes that have been experimentally studied are multicopper oxidases that use diphenols and related substances as donors, with oxygen as acceptor, and are known for their role in the enzymatic conversion of recalcitrant polysaccharides such as lignin [27]. ZC4RG08 is also noteworthy because it is the MAG that expresses the largest variety of ARGs among the MAGs here studied, suggesting it may be intrinsically resistant to several antibiotics; this characteristic has been reported for other members of the *Pseudomonas* genus [49].

Other noteworthy cluster 3 MAGs are ZC4RG13 (*Rhodothermus marinus*), ZC4RG21 (*Thermobifida fusca*), ZC4RG22 (Luteitaleales), and ZC4RG29 (Cyclobacteriaceae) (Fig. 5). During the late stages it is hypothesized that oxygen becomes more limited inside the composting pile and denitrification processes come into play (Supplementary Fig. 2). Accordingly, the above MAGs, plus ZC4RG26 (*Sphaerobacter thermophilus*), not included in any cluster, express genes annotated as nitrous-oxide reductase (including *nos*Z and *nos*D), which is the last step in denitrification, thus being evidence that these MAGs are able to perform the complete pathway (Table 2). The ability to utilize nitrous oxide (N_2_O) for anaerobic respiration might be crucial to improve efficiency of nitrogen utilization by bacteria in the composting process. Moreover, it is worth mentioning that N_2_O is a potent greenhouse gas, and microbial conversion of N_2_O to N_2_ is so far the unique sink known for N_2_O in the biosphere [50]. Anaerobic respiration using nitrous oxide is a widespread trait in prokaryotes, however not all denitrifiers encode this final step in denitrification [50]. As mentioned, one of the MAGs expressing nitrous oxide reductase is ZC4RG22, which was classified as a member of the Acidobacteriota phylum. To our knowledge this is the first report of a nitrous oxide reductase in this phylum [50].

By applying metabolic modeling methods, we were able to predict metabolic dependencies between MAGs. These results suggest that metabolic interactions in composting can be determined by complementary functions found in the genomes of producers and consumers. According to the Black Queen hypothesis [51], in order to increase fitness, one microbial population may lose genes related to a function when that function is already provided by another microbial population in the community. Therefore, the genomic differences between closely related strains are likely to be driven by local adaptation and coevolutionary interactions [51, 52]. This could explain why composting bacteria can present different metabolic dependencies compared with closely related strains that were isolated from other environments (Supplementary Table 14). Based on the predictions of compounds exchanged more frequently, our model suggests that major interactions were related to fermentation products (Supplementary Table 13). Dependencies on H^+^ exchange can be associated with hydrogenase activity (Table 2) and proton flux across membranes, which is related to ATP synthesis, pH homeostasis, and maintenance of solute gradients [53].

A metabolite transported into the extracellular environment as a waste product by one bacterium is often used by neighboring bacteria [51, 52]. This could also explain the O_2_ exchange flux identified (Supplementary Table 13). Oxygen can be a product derived from reactive oxygen species (ROS) detoxification systems, such as those encoded by chlorate-reducing bacteria via chloride dismutase [54]. Due to intense redox activity, ROS-detoxification is a vital function in the composting microbiome, as observed also by the overall profile of dominant functions detected in the ZC4 metatranscriptome dataset (Supplementary Table 15). Succinate also plays important roles in ROS management [55]. The higher exchange rates of succinate can also be associated with a potential demand for succinylation of cellulase enzymes, since it has been demonstrated that this process can enhance enzyme activity nearly twofold [56]. Thus, our data suggest a putative positive feedback loop in the composting microbiome, by which leak sugars from cellulose breakdown are shared from degraders to fermenters, and then fermenters return fermentation by-products such as succinate to degraders, and this can increase efficiency of cellulolytic enzymes. Another important detected pathway is related to the phosphate starvation response (Supplementary Table 15), which is also consistent with the dependency of phosphate exchange between MAGs (Supplementary Table 13).

Our model shows that ZC4RG13 (*Rhodothermus marinus*), ZC4RG04 (*Thermobispora bispora*), and ZC4RG20 (a novel Gammaproteobacteria species) are potential key players in the metabolic interactions during lignocellulose saccharification process (Supplementary Table 14). Although their genomes were recovered only from ZC4 metagenomes, our stringent genome recovery criteria may have prevented the recovery of these genomes in the datasets from the ZC3 composting cell. We do have evidence that these genomes were also present in the ZC3 composting metagenome (Supplementary Table 16).

Among the hypotheses generated in this work that could be verified experimentally, we mention the possible isolation of the Gammaproteobacteria bacterium (ZC4RG20), which presents the largest repertoire of AAs enzymes among analyzed MAGs, as well as the isolation of the ZC4 strains of *Thermobispora bispora*, *Rhodothermus marinus,* and fermentative bacteria that encode hydrogenases and are capable of exchanging fermentation by-products. These isolates could be tried as biomass degrading bacterial inoculants, and they might be particularly promising if they encode novel pathways towards efficient saccharification on industrial processes.

## Conclusions

With this work, we have added new knowledge on various aspects of the São Paulo Zoo composting process, on top of our previous work [1]. We now have solid hypotheses as to which bacteria are the main players in the process, and know almost all of their gene contents. Twenty-four of these bacteria are completely novel. We were able to get an idea of the temporal variation in abundance of each of these organisms throughout the composting process. This in turn enabled us to build a dynamic model of interactions of these bacteria, which is also a contribution to the molecular understanding of composting in general. The model, of course, is a hypothesis, and additional research will be needed to verify its predictions. Taken together, our results contribute to future research aiming at the engineering of efficient biomass-degrading thermophilic microbiomes.

## Methods

### Composting metagenomic and metatranscriptomic data

The composting metagenomic datasets on which this study is based have been described previously [1]. Briefly, the samples come from the composting facility at the São Paulo Zoo Park in the city of São Paulo, Brazil. Two composting cells were sampled: one called ZC3 and the other ZC4. For both, composting lasted 99 days at 60-70 °C most of the time. For ZC3, samples were collected on days 1, 30, 64, 78, and 99, and for ZC4 they were collected on days 1, 3, 7, 15, 30, 64, 67, 78, and 99 of the thermophilic composting process. A turning procedure was performed on day 65 for ZC3 and on day 63 for ZC4. DNA shotgun sequencing was done for all samples, and metatranscriptome sequencing was done for all ZC4 samples except day 64 sample [1].

### MAGs recovery workflow

Shotgun metagenomic reads from all samples from ZC3 and ZC4 composting cells were filtered and soft-trimmed (Quality values ≥12) using BBDuk from the BBTools package version 37.96 (https://jgi.doe.gov/data-and-tools/bbtools/). All shotgun reads used in the assemblies were obtained in a previous work [1]. Reads with length shorter than 80 bp were removed and the remaining reads were de novo assembled using metaSpades 3.12 (k-mer = 21,33,55,77,99,113,121,127, −-meta) [57]⁠⁠ (Supplementary Table 17). To obtain MAGs, the following steps were carried out, for each composting cell (ZC3 and ZC4) (Supplementary Fig. 3): 1) reads from all samples were assembled and the contigs binned with Metabat2 version 2.11 [58]; 2) reads from individual samples were assembled and the corresponding contigs were binned using Metabat2. After these two steps, only bins with completeness at least 50% and contamination at most 10% were kept for further processing, based on results from CheckM 1.0.12 [18]; 3) bins from individual samples (step 2) were compared with each other using Mash 2.1 [59]⁠, which allowed us to establish when the “same” bin occurred on different days (Mash distance at most 0.05); 4) for each “distinct” bin determined in step 3 its reads were reassembled and the results rebinned with Metabat2 [58]⁠ and MyCC version 1 [60]⁠; 5) bins from step (1) and those from step (4) were compared, again using Mash; 6) the MAGs selected for additional analyses were those distinct bins with best completeness and contamination results (when there was more than one bin for the same MAG), provided completeness was at least 80% and contamination at most 11% (Supplementary Fig. 3).

### Taxonomic assignment and genome comparisons

MAG taxonomic assignment was based on GTDB [26]. For those assignments that reached the species level, we carried out further comparisons with reference genomes of those species (whenever available, with complete genomes). These and other comparisons were done with the ANI tool (http://enve-omics.ce.gatech.edu/ani/) and with GGDC [61]. We refer to MAGs by their identifiers, providing in parenthesis the GTDB classification according to phylum, class, order, family, genus, or species. The comparison between our MAGs and those of the GEM catalog [22] was done with the tool fastANI 1.1 [62]. The GEM catalog makes available files with metadata information (the file is called genome_metadata, available in sql and tsv formats). These can be found at https://portal.nersc.gov/GEM/genomes/.

### Functional annotation

MAGs were annotated using the NCBI Prokaryotic Genome Annotation Pipeline [63]. Their protein-coding gene sequences were compared against the Clusters of Orthologous Groups (COGs) [64] database using rpsblast+ (blast version 2.9.0) [65], with a cut-off e-value of at most 10^− 5^. COG categories were assigned to the best hits with cdd2cog script (https://github.com/aleimba/bac-genomics-scripts/tree/master/cdd2cog). The amino acid sequences of the predicted coding sequences were classified for carbohydrate-active enzymes (CAZymes) [27]⁠ using dbCAN2 [66] using parameter values as described in http://bcb.unl.edu/dbCAN2/help.php. In the CAZymes database, enzymes are categorized in different classes and families, including key enzymes for lignocellulose degradation such as glycoside hydrolases (GHs) and auxiliary activities (AA), and the following complementary enzymes: glycosyltransferases (GTs), polysaccharide lyases (PLs), carbohydrate esterases (CEs) and carbohydrate-binding modules (CBMs). Genes were also classified using a set of HMMs for detecting other metabolic pathways of interest, such as genes involved in denitrification, sulfur metabolism, hydrogen metabolism, and oxygen metabolism [24, 67]. Evidence supporting MAGs with strictly anaerobic metabolism was obtained based on the consistency between the results provided by TRAITAR and gene classification as oxidases [68]. The global profile of functions in the metatranscriptomic dataset was determined using FMAP release v0.15 [69]⁠.

The presence of antibiotic resistance genes was analyzed in the MAGs by comparing protein-coding sequences against the CARD database (April 2019) [70]⁠ using the Resistance Gene Identifier via the CARD website RGI portal. We filtered out all results below 70% identity and 85% sequence coverage. antiSMASH 5.0 was used to find gene clusters involved in the biosynthesis of secondary metabolites [71].

#### Abundance and activity profiles of MAGs

The activity profile of MAGs across the metatranscriptomic datasets was obtained using the function *quant_bins* provided by metaWRAP 1.3 followed by normalization based on TPM (transcripts per kilobase million) [21]. Similarly, relative abundance of expressed genes was obtained by determining metatranscriptome reads that mapped to coding sequences using BEDTools 2.27.1 [72], followed by normalization based on TPM. We use the term *transcripts* to refer to MAG coding sequences to which metatranscriptome reads could be mapped.

### Correlation of MAGs based on their activity profiles

To identify patterns of co-occurring bacteria represented by MAGs in the ZC4 datasets, correlation analysis was performed based on relative abundance of MAGs and their transcripts, as described. We used CONET 1.1.1.beta [73] (Spearman r^2^ > 0.8) and the resulting graphs were visualized in Cytoscape 3.2.1 [74]⁠.

### Metabolic interaction models

Based on MAG co-occurrence patterns and the relative abundance of transcripts with annotation related to biomass degradation, denitrification, sulfur metabolism, hydrogen metabolism, and oxygen metabolism, we defined subsets of MAGs according to their importance in the different stages of the composting process. For each subset we built a metabolic interaction model using SMETANA 1.1.0 [75]⁠, based on genome-scale metabolic reconstructions that were obtained from files annotated in the PATRIC platform [76]. The results were submitted to Kbase [77]⁠ in order to run the *Build Metabolic Model* function, including the default option *gapfilling*, which relies on the ModelSEED Biochemistry Database [78]. With this method, metabolic genes were mapped onto biochemical reactions, and this information was integrated with information on stoichiometry reactions, subcellular localization, biomass composition, and estimation of thermodynamic feasibility, in order to produce a detailed stoichiometric model of metabolism at the genome scale. Metabolic dependency score calculated by SMETANA is normalized to a range between 0 and 1, meaning complete independency and complete dependency, respectively. Only strong metabolic dependencies; i.e., score = 1 [75]⁠ ⁠were considered.

## Supplementary Information

**Additional file 1.**

**Additional file 2.**

## Data Availability

The genome sequence and annotation of all MAGs described in this work are available from GenBank, and their accession numbers and permanent links are the following: **Table:** 

MAG_ID	BioSample	Accession	Link
ZC3RG01	SAMN08097825	PITW00000000	https://www.ncbi.nlm.nih.gov/nuccore/PITW00000000
ZC3RG02	SAMN08097829	PITX00000000	https://www.ncbi.nlm.nih.gov/nuccore/PITX00000000
ZC3RG03	SAMN08097823	PITV00000000	https://www.ncbi.nlm.nih.gov/nuccore/PITV00000000
ZC3RG04	SAMN08096851	PITU00000000	https://www.ncbi.nlm.nih.gov/nuccore/PITU00000000
ZC3RG05	SAMN08101807	PITY00000000	https://www.ncbi.nlm.nih.gov/nuccore/PITY00000000
ZC3RG06	SAMN08101822	PITZ00000000	https://www.ncbi.nlm.nih.gov/nuccore/PITZ00000000
ZC3RG07	SAMN08101823	PIUA00000000	https://www.ncbi.nlm.nih.gov/nuccore/PIUA00000000
ZC3RG08	SAMN08102237	PIUB00000000	https://www.ncbi.nlm.nih.gov/nuccore/PIUB00000000
ZC3RG09	SAMN09092868	QGSS00000000	https://www.ncbi.nlm.nih.gov/nuccore/QGSS00000000
ZC3RG10	SAMN09092869	QGSR00000000	https://www.ncbi.nlm.nih.gov/nuccore/QGSR00000000
ZC3RG11	SAMN09092870	QGSQ00000000	https://www.ncbi.nlm.nih.gov/nuccore/QGSQ00000000
ZC4RG01	SAMN08227010	PKQW00000000	https://www.ncbi.nlm.nih.gov/nuccore/PKQW00000000
ZC4RG02	SAMN08227009	PKQV00000000	https://www.ncbi.nlm.nih.gov/nuccore/PKQV00000000
ZC4RG03	SAMN08227008	PKQU00000000	https://www.ncbi.nlm.nih.gov/nuccore/PKQU00000000
ZC4RG04	SAMN08227007	PKQT00000000	https://www.ncbi.nlm.nih.gov/nuccore/PKQT00000000
ZC4RG05	SAMN08227006	PKQS00000000	https://www.ncbi.nlm.nih.gov/nuccore/PKQS00000000
ZC4RG06	SAMN08227005	PKQR00000000	https://www.ncbi.nlm.nih.gov/nuccore/PKQR00000000
ZC4RG07	SAMN08227012	PKQY00000000	https://www.ncbi.nlm.nih.gov/nuccore/PKQY00000000
ZC4RG08	SAMN08227013	PKQZ00000000	https://www.ncbi.nlm.nih.gov/nuccore/PKQZ00000000
ZC4RG09	SAMN08227011	PKQX00000000	https://www.ncbi.nlm.nih.gov/nuccore/PKQX00000000
ZC4RG10	SAMN08227015	PKRB00000000	https://www.ncbi.nlm.nih.gov/nuccore/PKRB00000000
ZC4RG11	SAMN08227014	PKRA00000000	https://www.ncbi.nlm.nih.gov/nuccore/PKRA00000000
ZC4RG12	SAMN08227016	PKRC00000000	https://www.ncbi.nlm.nih.gov/nuccore/PKRC00000000
ZC4RG13	SAMN08227017	PKRD00000000	https://www.ncbi.nlm.nih.gov/nuccore/PKRD00000000
ZC4RG14	SAMN08227018	PKRE00000000	https://www.ncbi.nlm.nih.gov/nuccore/PKRE00000000
ZC4RG15	SAMN08227019	PKRF00000000	https://www.ncbi.nlm.nih.gov/nuccore/PKRF00000000
ZC4RG16	SAMN09092792	QGVH00000000	https://www.ncbi.nlm.nih.gov/nuccore/QGVH00000000
ZC4RG17	SAMN08227020	PKRG00000000	https://www.ncbi.nlm.nih.gov/nuccore/PKRG00000000
ZC4RG18	SAMN08227022	PKRI00000000	https://www.ncbi.nlm.nih.gov/nuccore/PKRI00000000
ZC4RG19	SAMN08227021	PKRH00000000	https://www.ncbi.nlm.nih.gov/nuccore/PKRH00000000
ZC4RG20	SAMN08227023	PKRJ00000000	https://www.ncbi.nlm.nih.gov/nuccore/PKRJ00000000
ZC4RG21	SAMN09092793	QGVG00000000	https://www.ncbi.nlm.nih.gov/nuccore/QGVG00000000
ZC4RG22	SAMN09092794	QGVF00000000	https://www.ncbi.nlm.nih.gov/nuccore/QGVF00000000
ZC4RG23	SAMN09092795	QGVE00000000	https://www.ncbi.nlm.nih.gov/nuccore/QGVE00000000
ZC4RG24	SAMN09092796	QGVD00000000	https://www.ncbi.nlm.nih.gov/nuccore/QGVD00000000
ZC4RG25	SAMN09092797	QGVC00000000	https://www.ncbi.nlm.nih.gov/nuccore/QGVC00000000
ZC4RG26	SAMN09092798	QGVB00000000	https://www.ncbi.nlm.nih.gov/nuccore/QGVB00000000
ZC4RG27	SAMN09092799	QGVA00000000	https://www.ncbi.nlm.nih.gov/nuccore/QGVA00000000
ZC4RG28	SAMN09092800	QGUZ00000000	https://www.ncbi.nlm.nih.gov/nuccore/QGUZ00000000
ZC4RG29	SAMN09092801	QGUY00000000	https://www.ncbi.nlm.nih.gov/nuccore/QGUY00000000
ZC4RG30	SAMN09092802	QGUX00000000	https://www.ncbi.nlm.nih.gov/nuccore/QGUX00000000
ZC4RG31	SAMN09092803	QGUW00000000	https://www.ncbi.nlm.nih.gov/nuccore/QGUW00000000
ZC4RG32	SAMN09092804	QGUV00000000	https://www.ncbi.nlm.nih.gov/nuccore/QGUV00000000
ZC4RG33	SAMN09092805	QGUU00000000	https://www.ncbi.nlm.nih.gov/nuccore/QGUU00000000
ZC4RG34	SAMN09092806	QGUT00000000	https://www.ncbi.nlm.nih.gov/nuccore/QGUT00000000
ZC4RG35	SAMN09092807	QGUS00000000	https://www.ncbi.nlm.nih.gov/nuccore/QGUS00000000
ZC4RG36	SAMN09092808	QGUR00000000	https://www.ncbi.nlm.nih.gov/nuccore/QGUR00000000
ZC4RG37	SAMN09092809	QGUQ00000000	https://www.ncbi.nlm.nih.gov/nuccore/QGUQ00000000
ZC4RG38	SAMN09092810	QGUP00000000	https://www.ncbi.nlm.nih.gov/nuccore/QGUP00000000
ZC4RG39	SAMN09092811	QGUO00000000	https://www.ncbi.nlm.nih.gov/nuccore/QGUO00000000
ZC4RG40	SAMN09092812	QGUN00000000	https://www.ncbi.nlm.nih.gov/nuccore/QGUN00000000
ZC4RG41	SAMN09092813	QGUM00000000	https://www.ncbi.nlm.nih.gov/nuccore/QGUM00000000
ZC4RG42	SAMN09092814	QGUL00000000	https://www.ncbi.nlm.nih.gov/nuccore/QGUL00000000
ZC4RG43	SAMN09092815	QGUK00000000	https://www.ncbi.nlm.nih.gov/nuccore/QGUK00000000
ZC4RG44	SAMN09092816	QGUJ00000000	https://www.ncbi.nlm.nih.gov/nuccore/QGUJ00000000
ZC4RG45	SAMN09092817	QGUI00000000	https://www.ncbi.nlm.nih.gov/nuccore/QGUI00000000
ZC4RG46	SAMN09092818	QGUH00000000	https://www.ncbi.nlm.nih.gov/nuccore/QGUH00000000
ZC4RG47	SAMN09092819	QGUG00000000	https://www.ncbi.nlm.nih.gov/nuccore/QGUG00000000
ZC4RG48	SAMN09092820	QGUF00000000	https://www.ncbi.nlm.nih.gov/nuccore/QGUF00000000
ZC4RG49	SAMN09092821	QGUE00000000	https://www.ncbi.nlm.nih.gov/nuccore/QGUE00000000 The datasets supporting the conclusions of this article are included within the article (and its additional files).

## Abbreviations

AA: Auxiliary Activities
ANI: Average Nucleotide Identity
ARG: Antibiotic Resistance Gene
CAZy: Carbohydrate-Active enZYmes Database
CARD: Comprehensive Antibiotic Resistance Database
CBM: Carbohydrate-Binding Module
CE: Carbohydrate Esterase
COG: Cluster of Orthologous Group
DDH: DNA-to-DNA hybridization
GH: Glycoside Hydrolase
GT: Glycosyltransferase
HE: Highly Expressed
HMM: Hidden Markov Model
MAG: Metagenome-Assembled Genome
NCBI: National Center for Biotechnology Information
PL: Polysaccharide Lyase
TPM: Transcripts per kilobase million reads
