# Supplementary material for: Genome-resolved metagenome and metatranscriptome analyses of thermophilic composting reveal key bacterial players and their metabolic interactions
Source: BMC Genomics. 2021 Sep 10;22:652. doi: 10.1186/s12864-021-07957-9 (PMC8434746; doi:10.1186/s12864-021-07957-9)
Supplement: Supplementary file 2 — Additional file 2. [file 12864_2021_7957_MOESM2_ESM.pdf]

Supplementary Figures

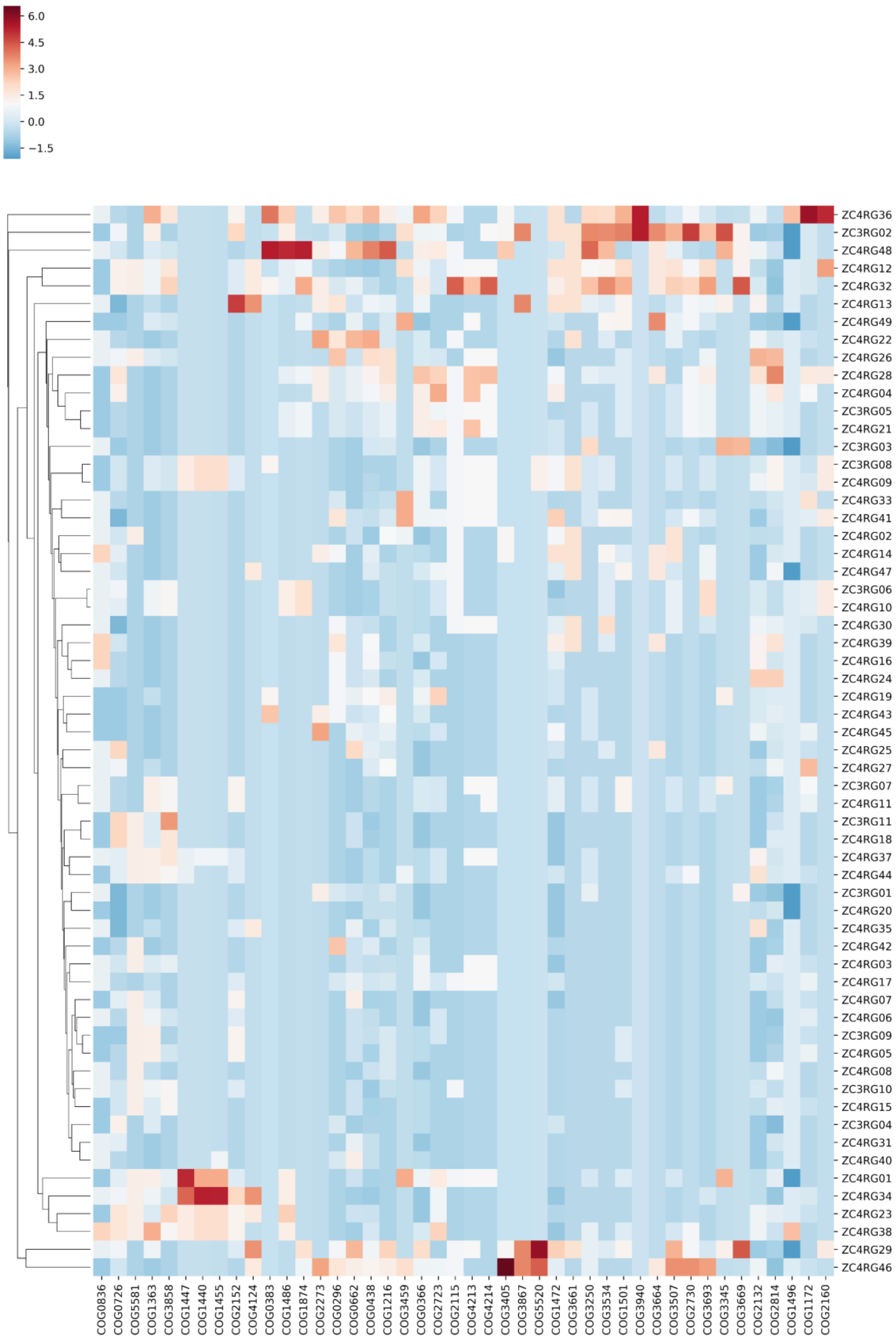

Supplementary Figure 1 - Hierarchical clustering of ZC3 and ZC4 MAGs. The heatmap is based on the number of COGs associated to lignocellulose breakdown. Abundant COGs are shown in red.

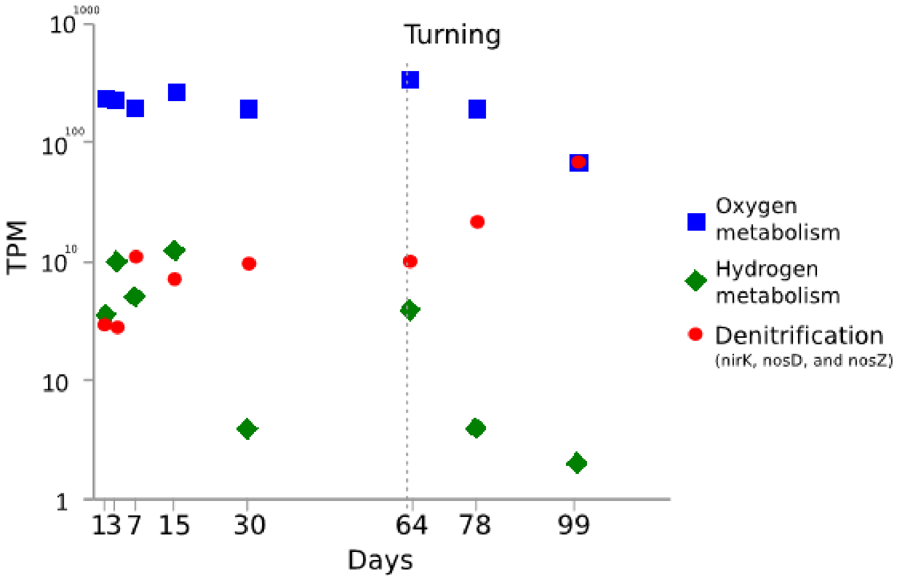

Supplementary Figure 2 – Variation in transcript abundance (TPM) of genes in MAGs that were associated to the indicated metabolic functions. Y-axis is in log scale.

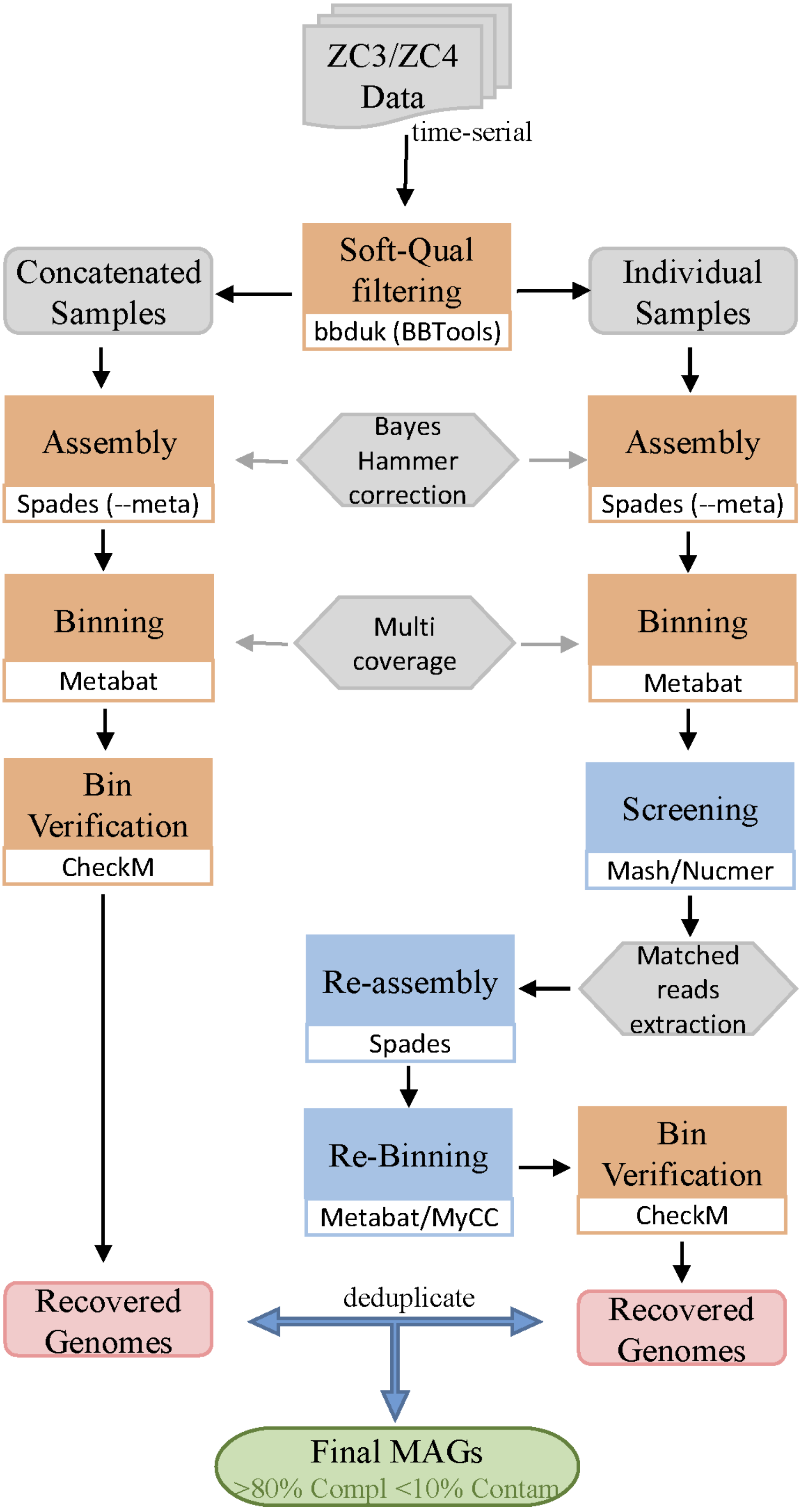

Supplementary Figure 3 – Workflow employed for recovering the ZC3 and ZC4 MAGs.
